# Risk to Nonparticipants in HIV Remission Studies With Treatment Interruption: A Symposium

**DOI:** 10.1093/infdis/jiz173

**Published:** 2019-07-02

**Authors:** Nir Eyal, Steven G Deeks

**Affiliations:** 1 Center for Population-Level Bioethics, Rutgers University, New Brunswick, New Jersey; 2 Department of Medicine, University of California, San Francisco

**Keywords:** HIV, research ethics, analytic treatment interruption, HIV cure-related studies, immunotherapy, pre-exposure prophylaxis

## Abstract

Ethical guidelines and recommendations for human subjects research typically focus on protecting the individuals who directly participate in that research. However, additional people, including sex partners of research participants, can also face harms and burdens from medical studies. In human immunodeficiency virus (HIV) cure–related research, a persistent ethical and practical challenge surrounds the use of analytical antiretroviral treatment interruptions. The challenge is usually discussed in relation to risks to study participants, but serious dangers accrue to nonparticipants, including sex partners of study participants. This multidisciplinary supplement relays the risks for nonparticipating sex partners in HIV cure–related studies and addresses the ethical dilemmas raised by these studies, with recommendations for researchers, advocates, sponsors, and oversight bodies.

Although a robust and reproducible measurement of the total body burden of replication-competent human immunodeficiency virus (HIV) is missing [1], dozens of HIV cure–related clinical trials have taken place [2]. In the absence of a viable biomarker for the reservoir, many clinical trialists are measuring the impact of their intervention by interrupting antiretroviral therapy (ART). These so-called analytic treatment interruption (ATI) protocols carry substantial risk to the participant [3–7]. However, because ART is highly efficacious in preventing transmission of HIV to sex partners [8], participants who stop treatment as part of a study pose an infection risk to their sex partners, who are not active study participants. This scenario raises further ethical and regulatory questions. Whereas ATIs might also have risked transmission to fetuses, given the broad agreement that transmission risks to fetuses are already too high, pregnant women are regularly excluded from ATI studies [9, 10]. This supplement focuses on the risks to the sex partners of participants in cure-related studies that include an ATI.

The risk that an ATI poses to any sex partner depends on the details of the ATI, which, in turn, depend on the overall objectives of the intervention being studied. For studies that seek to dramatically reduce or eliminate the reservoir, a highly monitored “pause” of ART to assess success (ie, cure) may suffice [11, 12]. If a participant is carefully followed up with frequent (at least weekly) viral load monitoring and if ART is resumed as soon as virus is detected (thus ruling out cure), it is theoretically possible to resume therapy before viremia peaks to a level that poses a substantial and sustained risk to sex partners. One pilot study suggests that such a study is possible, even if logistically challenging [12].

The risk to sex partners (as well as to participants) is much higher in ATI studies that require measurement of the steady-state viral load set point. The goal of the intervention in these studies is generally to achieve durable posttreatment control of a replication-competent reservoir (ie, “remission”) [13]. Based on observations made in natural infection and in some of the posttreatment control cohorts, a period of acute viremia will likely occur before the immune system is able to respond and regain control. Plasma HIV RNA levels might need to remain well over 10 000 copies RNA/mL for a few weeks before control is gradually achieved [14, 15]. The risk of such studies is much greater than those in which ART is resumed once a rebound is detected.

The potential need to allow for a period of acute viremia before virus control is obtained was well illustrated in a study of simian immunodeficiency virus (SIV)-infected macaques on ART who received a combination of a therapeutic vaccine and a TLR7 agonist (Figure 1) [16]. When ART was interrupted, virus rebounded sharply within 2 to 3 weeks, peaking at over 10^4^ copies/ml and remaining high for several weeks before subsequent control was obtained. Had treatment been resumed immediately upon recrudescence and before or during the period of high viremia, then the success that eventually became evident would not have been observed. Similar periods of viremia before control was obtained was observed in studies of broadly neutralizing antibodies [17]. Acute viremia that can be high has also been observed in individuals who appear to have achieved treatment-free remission as a consequence of ART (ie, “post-treatment controllers”) [15, 18].

**Figure 1.**
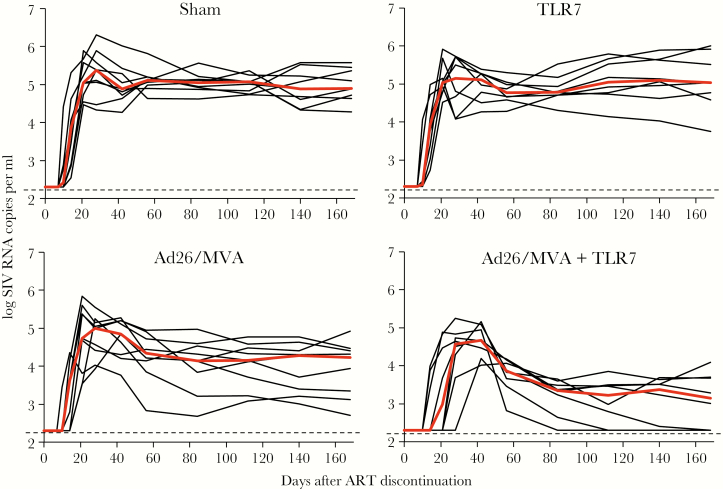
A transient period of high-level viremia that may last weeks may be necessary before the effects of an immunotherapy become apparent. In a study of a therapeutic vaccine (recombinant adenovirus serotype 26 prime followed by modified vaccinia Ankara boost, Ad26/MVA) and a toll-like receptor agonist (TLR7) preformed in 40 SIV-infected macaques on antiretroviral therapy, those who received both therapies experienced a period of high-level viremia for several weeks before immune-mediated virus control was achieved. Many of the interventions being studied in HIV-infected people have treatment interruptions which allow for similar periods of viremia [16].

Current ethics of HIV cure–related research examines ATI risks primarily for the participant and not for their sex partners [5, 12, 14]. Our supplement reports on a case in which the risk to a sex partner materialized. The ethical issues that the latter risk poses are especially hard. Study nonparticipants at risk from sexual acquisition normally remain uninformed of this risk and are not advised on how to reduce this risk. Preexposure prophylaxis (PrEP) is not always offered. Partners are not always monitored for infection and may receive no advice or support should it occur. For a study participant, part of the justification of the risk (though not the main part) [6] is that, in some studies, there may be a possibility of a remission or cure—a direct benefit. By contrast, ATI (and, normally, the study in general) provides no possible direct benefit to the partner.

This supplement expands current understanding of the risks among nonparticipants in HIV cure–related studies and explores the ethics and proper oversight of these studies. In particular, how might researchers reduce risks to study nonparticipants while respecting study participants’ confidentiality rights? Should study investigators be required to provide participants safer-sex counseling, and should a candidate participant’s refusal to undergo counseling or close monitoring exclude her or him from participation in remission studies that include an ATI? Should investigators be obligated to provide participants’ sex partners in stable relationships with services, ranging from counseling to PrEP? Should the partners need to assent to their partner’s participation in the study? Would it make sense to exclude candidate participants on the basis of partners in stable relationships characteristics, such as a preference for unsafe sex? In the case of potentially less stable sex partnerships, should researchers seek “community consent” to studies? Is some research teams’ and sponsors’ seeming reluctance to take protective measures for fear of being seen to take ownership over any HIV infections misguided?

Our supplement starts with a report from HIV remission researchers Jean-Daniel Lelièvre and Laurent Hocqueloux that describes unintended transmission of HIV to a sex partner in a stable relationship in their group’s therapeutic vaccine study. It continues with perspectives from ethicists. One, by Nir Eyal, evaluates different strategies for mitigating and justifying the risk of partner infection in remission studies with an ATI. Another, by Liza Dawson, examines how a relational approach to bioethics would address the study risks to nonparticipants. The supplement then addresses specific measures to prevent transmission to sex partners in HIV cure–related studies involving an ATI. HIV remission researcher Jean-Daniel Lelièvre focuses on one protection: offering PrEP to participants’ sex partners is usually sensible, but it raises complications and should be introduced with care. A commentary by Eyal and Monica Magalhaes assesses the case for one extreme protection: isolation for remission-study participants during ATIs.

Remarkably, only a few of the recommended measures are part of either current or completed immunotherapeutic HIV remission studies that include ATIs. A final commentary by Eyal seeks to clear one barrier to greater protection for sex partners in studies that include ATIs: the false impression that increased protection of sex partners would make sponsors or research institutes liable if the partners acquire infection.

While the supplement’s general focus is on the ethical response to risks to nonparticipants in HIV cure–related studies with an ATI, we would be remiss without mentioning the continuing disagreement on the scientific unavoidability of ATIs in these studies. The supplement concludes with a debate between HIV cure and remission researchers David Margolis and Steven Deeks. Margolis doubts the need for use of ATIs in many cure-related studies. Deeks argues that, with appropriate protections, ATIs remain unavoidable. All in all, these articles point to the need for both standards and deeper ethical deliberation on governing infection risks to nonparticipants in HIV remission studies with an ATI [19–23].

## Notes

Presented in part: Medical Study Risks to Nonparticipants: Ethical Considerations Workshop, Boston, Massachusetts, 11–12 June 2017.

## Notes

***Financial support.*** This supplement is sponsored by NIH: National Institute Allergy and Infectious Diseases [grant number 1 R01 AI114617-01A1 (HIV cure studies: risk, risk perception, and ethics)].

***Potential conflicts of interest.*** S. G. D. receives grant support from Gilead, Merck, and ViiV and is an advisor for Enochian Biosciences and BryoLogyx. N. E. certifies no potential conflicts of interest.

Both authors have submitted the ICMJE Form for Disclosure of Potential Conflicts of Interest. Conflicts that the editors consider relevant to the content of the manuscript have been disclosed.
